# Cost-Effective Modeling of Thromboembolic Chemoprophylaxis for Total Ankle Arthroplasty

**DOI:** 10.1177/10711007221112922

**Published:** 2022-07-28

**Authors:** Brandon J. Martinazzi, Gregory J. Kirchner, Christopher M. Stauch, F. Jeffrey Lorenz, Kristen M. Manto, Vincenzo Bonaddio, Zachary Koroneos, Michael C. Aynardi

**Affiliations:** 1 Department of Orthopaedics & Rehabilitation, Penn State Health, Milton S. Hershey Medical Center, Hershey, PA, USA

**Keywords:** VTE, cost-effectiveness, economic modeling, ankle arthroplasty

## Abstract

**Background:**

Symptomatic venous thromboembolism (VTE) following total ankle arthroplasty (TAA) can cause substantial morbidity and mortality. To prevent this complication, surgeons often prescribe postoperative chemoprophylaxis. However, much controversy exists regarding the efficacy of chemoprophylaxis because of the limited studies exploring its use. Furthermore, even less is known about its cost-effectiveness. Therefore, this study sought to determine the cost-effectiveness of commonly prescribed chemoprophylactic agents using a break-even analysis economic model.

**Methods:**

The literature was searched, and an online database was used to identify patients who developed a symptomatic VTE after undergoing TAA. Our institutional records were used to estimate the cost of treating a symptomatic VTE, and an online drug database was used to obtain the cost of commonly prescribed chemoprophylactic agents. A break-even analysis was then performed to determine the final break-even rate necessary to make a drug cost-effective.

**Results:**

The low and high rates of symptomatic VTE were determined to be 0.46% and 9.8%. From 2011 to 2021, a total of 3455 patients underwent total ankle arthroplasty. Of these patients, 16 developed a postoperative symptomatic VTE (1.01%). Aspirin 81 mg was cost-effective if the initial symptomatic VTE rates decreased by an absolute risk reduction (ARR) of 0.0003% (NNT = 31 357). Aspirin 325 mg was also cost-effective if the initial rates decreased by an ARR 0.02% (NNT = 5807). Likewise, warfarin (5 mg) was cost-effective at all initial rates with an ARR of 0.02% (NNT = 4480). In contrast, enoxaparin (40 mg) and rivaroxaban (20 mg) were only cost-effective at higher initial symptomatic VTE rates with ARRs of 1.48% (NNT = 68) and 5.36% (NNT = 19). Additional analyses demonstrated that enoxaparin (40 mg) and rivaroxaban (20 mg) become cost-effective when costs of treating a symptomatic VTE are higher than our estimates.

**Conclusion:**

Chemoprophylaxis following TAA can be cost-effective. A tailored approach to VTE prophylaxis with cost-effectiveness in mind may be beneficial to the patient and health system.

**Level of Evidence:** Level IV,economic analysis.

## Introduction

Symptomatic venous thromboembolism (VTE), which includes deep venous thrombosis (DVT) and pulmonary embolism (PE), occurs at a rate ranging from 0.46% to 9.8% following total ankle arthroplasty (TAA).^1,2,4,10^ The consequences of symptomatic VTE following TAA can be devastating both medically and economically. For example, the 1-month mortality following the diagnosis of DVT or PE is as high as 6% and 12%, respectively.^13^ Furthermore, a symptomatic VTE during admission can add up to $17 000 in hospital expenses, and total cost of treatment for a symptomatic VTE has been reported to be $33 000 during the first year following the event.^11^ Despite these added burdens to the patient and health system as a result of symptomatic VTE, much debate continues regarding the merits of VTE chemoprophylaxis in foot and ankle surgery.^6,12^

Limited evidence of the efficacy of VTE chemoprophylaxis following TAA is the primary cause of continued debate regarding its use. Although there is an abundance of literature on chemoprophylactic agents such as aspirin and enoxaparin following arthroplasty of the hip or knee, similar quality literature is simply not available to guide foot and ankle surgeons in their practice.^3,7^ This is primarily due to the generally lower rates of symptomatic VTE following TAA relative to other procedures. However, many VTEs are clinically silent and, therefore, likely higher than what has been reported. With symptomatic VTE rates cited as low as 0.46%, generating high-quality studies with adequate power are not practical.^1^ However, even at lower rates, symptomatic VTE remains a major complication following TAA.

Although the effectiveness of VTE chemoprophylaxis following TAA remains unclear, the goal of this study was to determine if the use of VTE chemoprophylaxis following TAA is justifiable from an economic standpoint. Understanding the economic practicality of common VTE chemoprophylactic agents may help ankle replacement surgeons determine whether VTE chemoprophylaxis is beneficial to their patients and practice. Given the varying costs associated with the common VTE prophylactic agents, we hypothesized that agents such as aspirin, warfarin, and enoxaparin would be more cost-effective than newer agents such as rivaroxaban.

## Methods

**Figure 1.**
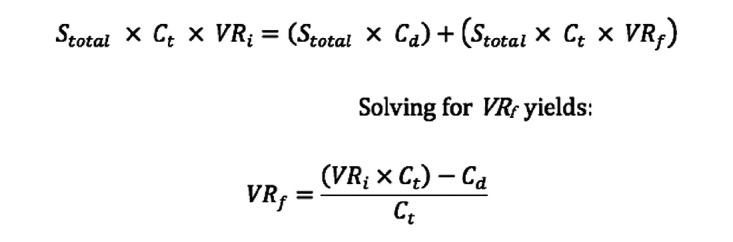
Equation used to calculate break-even VTE rate. Where: *S_total_* = total annual surgeries; *C_t_* = total cost of treating a VTE; *C_d_* = cost of drug(s); *VR_i_* = initial VTE rate; *VR_f_* = breakeven VTE rate. Source: Adapted from Hatch et al.^9^

A “break-even” analysis was performed using a modified equation initially described by Hatch et al^9^ (Figure 1). This equation produces the final break-even rate necessary to make an intervention cost-effective. Subtracting the final break-even rate from the initial rate gives the absolute risk reduction (ARR), which is the percentage by which an intervention must reduce the initial symptomatic VTE rate in order to justify its use economically. Using the ARR, we then calculated the number needed to treat (NNT). In our study, the NNT represents how many cases could be performed while only preventing a single symptomatic VTE in order to “break-even” on cost.

Our institution’s purchasing records were used to estimate the cost associated with treating a VTE. An online drug database (GoodRX) was then used to obtain the average retail cost for a 1-month course of daily aspirin (81 mg), aspirin (325 mg), enoxaparin (40 mg), rivaroxaban (20 mg), and warfarin (5 mg).^8^ This database is a free online tool that patients can use to obtain their prescriptions for the lowest possible price. We chose a 1-month course because this is routinely used for VTE prophylaxis following TAA at our institution. Additionally, we followed the same rationale for the dosage of drugs used. Of note, the product cost of International Normalized Ratio (INR) monitoring for warfarin has been shown to vary from as low as $6.19 to as high as $145.70.^5^ As such, we performed an additional analysis using the lowest cost of INR monitoring.

The literature was then searched to obtain the reported rates of symptomatic VTE following TAA. In an effort to establish an objective incidence of symptomatic VTE in patients without VTE prophylaxis, the TriNetX Research Database was queried using the *International Classification of Diseases* codes I26 (Pulmonary embolism), I82.62 (Acute embolism and thrombosis of deep veins of upper extremity), and I82.64 (Acute embolism and thrombosis of deep veins of lower extremity) and *Current Procedural Terminology* code 1014588 (Arthroplasty, Ankle) to determine the VTE rate within 4 weeks of surgery. This database provides access to electronic medical records for approximately 83 million patients from more than 50 health care organizations. All data present in the database are deidentified patient data, which allowed this study to be exempt from institutional review board approval. Demographics of the patient population were also evaluated (Table 1). All patients, regardless of age, sex, race, or ethnicity who underwent TAA and did not receive VTE prophylaxis postoperatively were included in our final analysis. Patients who underwent TAA and received postoperative VTE prophylaxis were excluded.

**Table 1.** Demographics of TriNetX Patient Population.

Clinical Characteristic	
Mean age, y	38.3 ± 16.3
Sex, n (%)	
Female	47 233 (48)
Male	50 160 (51)
Unknown	1337 (1)
Ethnicity	
Not Hispanic or Latino	70 809 (72)
Hispanic or Latino	19 343 (19)
Unknown ethnicity	8578 (9)
Race	
White	65 560 (66)
Black or African American	18 262 (19)
Asian	12 278 (12)
American Indian or Alaskan Native	1993 (2)
Native Hawaiian or Other Pacific Islander	490 (1)
Unknown	147 (<1)

## Results

The product costs for a 1-month supply of once-daily aspirin (81 mg), aspirin (325 mg), enoxaparin (40 mg), rivaroxaban (20 mg), and warfarin (5 mg) were found to be $0.30, $1.62, $138.77, $504.23, and $2.10, respectively (Table 2). These were the lowest prices for a full month’s supply that a patient could obtain if they used GoodRx. The estimated cost of treating a symptomatic VTE at our institution was $9407. The low and high rates of symptomatic VTE obtained from the literature were determined to be 0.46% and 9.8%. Using the TriNetX Research Database, from 2011 to 2021, a total of 3455 patients underwent total ankle arthroplasty. Of these patients, 1577 did not receive chemoprophylaxis and 16 (16/1577 = 1.01%) were identified with a symptomatic VTE. At the product cost obtained, aspirin 81 mg was found to be cost-effective at the low, TriNetX, and high rate of symptomatic VTE if the initial rate decreased by an ARR of 0.0003% (NNT=31 357). Aspirin 325 mg was also cost-effective at all 3 initial rates with an ARR 0.02% (NNT = 5807). Likewise, warfarin (5 mg) was cost-effective at all 3 initial rates with an ARR of 0.02% (NNT = 4480). Additionally, warfarin remained cost-effective when factoring in the lowest cost of INR monitoring (NNT = 1135) (Table 3). In contrast, cost-effectiveness was eliminated at the low and TriNetX symptomatic VTE rate for enoxaparin (40 mg) and rivaroxaban (20 mg) because the final VTE rate exceeded the initial rate. Enoxaparin (40 mg) maintained cost-effectiveness at the high initial symptomatic VTE rate with an ARR of 1.48% (NNT = 68). Similarly, rivaroxaban (20 mg) was also cost-effective at the high initial symptomatic VTE rate with an ARR of 5.36% (NNT = 19) (Table 3). Additional analyses demonstrated that enoxaparin (40 mg) and rivaroxaban (20 mg) become cost-effective when costs of treating a symptomatic VTE are higher (Table 4).

**Table 2.** Cost of Common Chemoprophylactic Agents.

Drug	Dosing (mg)	Route of Administration	Average Retail Price, $
Aspirin	81	By mouth	0.30
Aspirin	325	By mouth	1.62
Enoxaparin	40	Subcutaneous	138.77
Rivaroxaban	20	By mouth	504.23
Warfarin	5	By mouth	2.10

**Table 3.** Cost-effectiveness of Chemoprophylactic Agents at Varying Initial VTE Rates.

Drug (mg)	Initial VTE Rate, %	Final VTE Rate, %	ARR, %	NNT
Aspirin 81	0.46	0.457	0.003	31 357
	1.01	1.017	0.003	31 357
	9.80	9.80	0.003	31 357
Aspirin 325	0.46	0.44	0.02	5807
	1.01	1.00	0.02	5807
	9.80	9.78	0.02	5807
Enoxaparin 40	0.46	–1.02	1.48	68
	1.01	–0.46	1.48	68
	9.80	8.32	1.48	68
Rivaroxaban 20	0.46	–4.90	5.36	19
	1.01	–4.34	5.36	19
	9.80	4.44	5.36	19
Warfarin 5	0.46	0.44	0.02	4480
	1.01	1.00	0.02	4480
	9.80	9.78	0.02	4480
Warfarin 5 + INR^a^	0.46	0.37	0.09	1135
	1.01	0.92	0.09	1135
	9.80	9.71	0.09	1135

Abbreviations: ARR, absolute risk reduction; INR, international normalized ratio; NNT, number needed to treat; VTE, venous thromboembolism.

a Assumes cost of INR monitoring for warfarin to be $6.19.

**Table 4.** More Expensive Drugs Become Cost-Effective at Higher Cost Associated With Treating VTE.

Drug (mg)	Initial VTE Rate, %	Cost of Treating VTE	Final VTE Rate, %	ARR, %	NNT
Enoxaparin 40	0.46	$9407.00	–1.015	1.48	68
	0.46	$30 000.00	–0.003	0.46	216
	0.46	$50 000.00	0.182	0.28	360
	0.46	$70 000.00	0.262	0.20	504
	0.46	$90 000.00	0.306	0.15	649
	0.46	$110 000.00	0.334	0.13	793
Rivaroxaban 20	0.46	$9407.00	–4.900	5.36	19
	0.46	$30 000.00	–1.221	1.68	59
	0.46	$50 000.00	–0.548	1.01	99
	0.46	$70 000.00	–0.260	0.72	139
	0.46	$90 000.00	–0.100	0.56	178
	0.46	$110 000.00	0.002	0.46	218

Abbreviations: ARR, absolute risk reduction; NNT, number needed to treat; VTE, venous thromboembolism.

## Discussion

The use of chemoprophylaxis following TAA continues to be debated.^12^ This likely stems from the wide range of symptomatic VTE rates reported in the literature, and the challenge associated with performing adequately powered studies of VTE chemoprophylaxis efficacy following TAA.^1,2,4,10^ Furthermore, performing prospective randomized controlled trials has the potential to place patients at an increased risk for developing a VTE in those who are undertreated. Moreover, one must also consider the potential of bleeding complications in patients who are overtreated. However, despite these difficulties, it is still important that we consider the economic burden of VTE on the healthcare system, as well as the added financial strain sustained by patients who develop VTE or those who are prescribed medication unnecessarily. This break-even analysis determined that aspirin and warfarin are highly cost-effective, whereas enoxaparin and rivaroxaban are cost-effective only at the highest cited rates of symptomatic VTE following TAA.

Although our study provides a simple conceptual economic model, it does have several flaws. First, the cost of the drugs that we used are likely to vary among institutions and geographical location. We chose to use an online GoodRx database as a way to represent some of the less expensive options for purchase. Moreover, at our institution, our senior surgeon traditionally prescribes chemoprophylaxis for up to 1 month following TAA. This too will likely vary by surgeon and will, therefore, affect the cost of the drug. For example, the total cost of a drug will differ for a surgeon who prescribes a form of chemoprophylaxis for only 2 weeks, as opposed to 1 month. Second, this study generalizes the procedure of TAA. In reality, there are multiple different types of implants that can be used during TAA that could affect duration of surgery and length of recovery, all of which could impact the incidence of developing a symptomatic VTE. Third, one could argue that there is some subjectivity in the incidence of symptomatic range that we selected. While we tried to objectify this information with the use of the TriNetX research network, large databases are based on *International Classification of Diseases* and *Current Procedural Terminology* codes, which rely on accurate coding of the outcomes of interest (Online Appendix 1). These codes fail to identify asymptomatic VTEs and do not account for unrecognized VTEs that occurred but not recorded in the TriNetX research network data set. Thus, it is reasonable to assume our TriNetX symptomatic VTE incidence may fail to capture the true incidence within the population.

Finally, economic modeling does not include individual patient information and looks instead at the larger demographic picture while making several assumptions. Chief among these assumptions is that all patients are receiving chemoprophylaxis following TAA, which is unlikely to be true. Furthermore, our study does not consider the cost of treating chemoprophylaxis bleeding complications or other indirect financial costs incurred by the patient during VTE treatment. Additionally, our study assumes that the cost of drugs, treatment for a symptomatic VTE, and the rate of symptomatic VTE are subject to change based on patient characteristics, the practicing surgeon, and geographic location. Despite these assumptions, a major advantage of our formula is that foot and ankle surgeons can use this equation to determine cost-effectiveness using their own patient population and regional pricing. Furthermore, our work adds to the limited pool of studies exploring VTE prophylaxis in foot and ankle surgery.

There are several important considerations derived from our “break-even” analysis. First, if we assume that the actual rate of symptomatic VTE following TAA is low, the major determinant of cost-effectiveness was the cost of the drug itself. At the low reported symptomatic VTE rate, only aspirin (81 and 325 mg) and warfarin (5 mg) were able to maintain cost-effectiveness. Likewise, including the lowest cost of INR monitoring for warfarin did not affect its ability to remain cost-effective. However, if the cost of INR monitoring is higher than what we used in our calculations, warfarin may lose its ability to remain cost-effective. This is an important point to consider because if a drug can be purchased for a lower price, it increases its chance of becoming cost-effective. Second, our study evaluates these drugs at varying initial symptomatic VTE rates in an effort to reflect the true unknown incidence in the general population. Therefore, we demonstrate that at higher initial symptomatic VTE rates, the more expensive rivaroxaban and enoxaparin can be cost-effective. Third, we show that the more expensive drugs (enoxaparin and rivaroxaban) are cost-effective at the lowest rate of symptomatic VTE if the cost of treating a symptomatic VTE complication is higher than what we estimated at our institution. Thus, in situations where patients may experience longer hospitalizations if they develop symptomatic VTE, and thus incur higher cost of treatment, both enoxaparin and rivaroxaban can be cost-effective even if the initial symptomatic VTE rate is as low as 0.46%. Finally, the modified Hatch et al^9^ equation yields theoretical ARRs that each of these drugs would need to achieve in order for them to be cost-effective. Therefore, if future studies determine actual ARRs, our data could be used as comparison to establish cost-effectiveness.

The utility of this “break-even” analysis is that it provides a simple way to determine the economic viability of these commonly used prophylactic agents. For example, assuming the hypothetical ARR of aspirin (81 mg) is 0.0003%, 31 357 patients would need to be treated to prevent a single symptomatic VTE. To determine the same result in a clinical trial, using a power analysis, the sample size would need to be 68 295 111 111, assuming a *P* <.05 and power equal to 80%. Therefore, our model provides data that would be unobtainable in a clinical study.

Based on our results, we believe that chemoprophylaxis can be cost-effective for the prevention of symptomatic VTE in TAA. Despite the ongoing controversy surrounding the use of these agents in foot and ankle surgery, chemoprophylaxis has successfully reduced the rates of symptomatic VTE in other areas of orthopaedics.^7^

Over time, TAA techniques and implant design have undergone enormous improvements, which has resulted in increased interest in perioperative patient optimization as a primary target for improving short-term outcomes following TAA. Current understanding of the clinical benefit of VTE prophylaxis following TAA is limited; however, current literature reflects an increased interest in VTE prophylaxis following TAA. As surgeons consider the various risks and benefits of VTE prophylaxis, particularly with the perspective of universal or near-universal application, the cost-effectiveness of the chemoprophylactic agents is certainly a factor that should be considered—as it can have economic consequences for patients, practices, and health systems alike. Ultimately, the decision to prescribe chemoprophylaxis should be multifactorial. Surgeons can use the presented formula as a contributing tool to determine the best chemoprophylaxis for their patients at this time. For example, after considering our studies’ results, a surgeon can investigate their own VTE rate and cost of treating a symptomatic VTE within their practice to determine if the drugs they are prescribing are economically justifiable. Going further, as future research will undoubtedly reveal new risks and benefits of certain chemoprophylactic agents, surgeons can return to this formula for reconsideration of the economics of chemoprophylactic agents for their patients.

It is important to note that we are not suggesting for or against the use of chemoprophylaxis or suggesting that one agent is superior to the other, we are simply providing an objective cost-effective analysis to further broaden the knowledge of cost associated with unwanted complications in TAA.

## Supplemental Material

sj-docx-1-fai-10.1177_10711007221112922 – Supplemental material for Cost-Effective Modeling of Thromboembolic Chemoprophylaxis for Total Ankle Arthroplasty Click here for additional data file. Supplemental material, sj-docx-1-fai-10.1177_10711007221112922 for Cost-Effective Modeling of Thromboembolic Chemoprophylaxis for Total Ankle Arthroplasty by Brandon J. Martinazzi, Gregory J. Kirchner, Christopher M. Stauch, F. Jeffrey Lorenz, Kirsten M. Manto, Vincenzo Bonaddio, Zachary Koroneos and Michael C. Aynardi in Foot & Ankle International

sj-pdf-2-fai-10.1177_10711007221112922 – Supplemental material for Cost-Effective Modeling of Thromboembolic Chemoprophylaxis for Total Ankle Arthroplasty Click here for additional data file. Supplemental material, sj-pdf-2-fai-10.1177_10711007221112922 for Cost-Effective Modeling of Thromboembolic Chemoprophylaxis for Total Ankle Arthroplasty by Brandon J. Martinazzi, Gregory J. Kirchner, Christopher M. Stauch, F. Jeffrey Lorenz, Kirsten M. Manto, Vincenzo Bonaddio, Zachary Koroneos and Michael C. Aynardi in Foot & Ankle International
