# Supplementary material for: Cost-Effective Modeling of Thromboembolic Chemoprophylaxis for Total Ankle Arthroplasty
Source: Foot Ankle Int. 2022 Jul 28;43(10):1379–84. doi: 10.1177/10711007221112922 (PMC9527361; doi:10.1177/10711007221112922)
Supplement: sj-docx-1-fai-10.1177_10711007221112922 – Supplemental material for Cost-Effective Modeling of Thromboembolic Chemoprophylaxis for Total Ankle Arthroplasty [file sj-docx-1-fai-10.1177_10711007221112922.docx]

**Appendix 1**

The TriNetX research network is a global federated research network that provides access to electronic medical records from more than 120 healthcare organizations across 19 countries. All data within the network is de-identified patient information. The network includes access to diagnoses, medications, procedures, genomics, and laboratory values, while providing information about age, sex, race, and ethnicity. The network excludes hospital expenses, non-hospital expenses, personal patient information, and information about functional outcomes. For example, no information can be obtained regarding a return to work or regular activity.

The main concern with the TriNetX research network is similar to the limitations encountered when using other large databases. These include a population limited to participating institutions and the need for accurate coding. Furthermore, there is always the concern regarding the quality of data in the network; however, TriNetX has developed a systematic method for evaluating data quality through regular refreshes to ensure accuracy. More information regarding the network can be found here: https://trinetx.com/
